# A Novel Anomaly-Based Intrusion Detection Model Using PSOGWO-Optimized BP Neural Network and GA-Based Feature Selection

**DOI:** 10.3390/s22239318

**Published:** 2022-11-30

**Authors:** Saeid Sheikhi, Panos Kostakos

**Affiliations:** Center for Ubiquitous Computing, University of Oulu, 90570 Oulu, Finland

**Keywords:** network intrusion, network intrusion detection, anomaly detection, cybersecurity, machine learning

## Abstract

Intrusion detection systems (IDS) are crucial for network security because they enable detection of and response to malicious traffic. However, as next-generation communications networks become increasingly diversified and interconnected, intrusion detection systems are confronted with dimensionality difficulties. Prior works have shown that high-dimensional datasets that simulate real-world network data increase the complexity and processing time of IDS system training and testing, while irrelevant features waste resources and reduce the detection rate. In this paper, a new intrusion detection model is presented which uses a genetic algorithm (GA) for feature selection and optimization algorithms for gradient descent. First, the GA-based method is used to select a set of highly correlated features from the NSL-KDD dataset that can significantly improve the detection ability of the proposed model. A Back-Propagation Neural Network (BPNN) is then trained using the HPSOGWO method, a hybrid combination of the Particle Swarm Optimization (PSO) and Grey Wolf Optimization (GWO) algorithms. Finally, the hybrid HPSOGWO-BPNN algorithm is used to solve binary and multi-class classification problems on the NSL-KDD dataset. The experimental outcomes demonstrate that the proposed model achieves better performance than other techniques in terms of accuracy, with a lower error rate and better ability to detect different types of attacks.

## 1. Introduction

With the rapid growth of the internet, the security of networks and systems is becoming more critical for organizations, companies, and individuals, indicating the urgent need for secure, robust, and trustworthy communications. In general, any unauthorized access to computer networks or systems is referred to as an intrusion [1]. Many security techniques have been studied and developed over the past decades for the protection of networks, such as cryptography, firewalls, and intrusion detection systems [2]. Among them, intrusion detection systems (IDS) are considered the most effective way to defend against dynamic and complex malicious behaviors [3]. An intrusion detection system monitors network activity and determines whether it is abnormal or normal [4]. The primary aim of an IDS is to detect and report intrusion or security events in the system or networks to the system administrator. These systems are applied as security supplements alongside firewalls.

Intrusion detection approaches are divided into two principal types: misuse detection systems (or signature matching) and anomaly detection systems [5]. Misuse detection systems identify attacks based on attack signatures and the parameters of system vulnerabilities. This is accomplished by comparing potential intrusion cases to previously recognized patterns. As such, this approach is obviously unable to detect unknown or emerging attacks. Anomaly intrusion detection systems, on the other hand, work based on typical behavior patterns and use them to recognize any network activity that deviates from the norm. More specifically, the anomaly mechanism identifies intrusion patterns by comparing analytical data acquired from regular use of the system [6].

Machine learning (ML) methods can be used to create a robust IDS, as they may be deployed to detect intrusions and make generalizations in specific network environments [7]. In most cases, there are several challenges to implementing an effective custom IDS method. The inherent implementation issues can be classified into different sets of problems based on the system’s usability, ability, and accuracy [6]. Nonetheless, intrusion detection systems based on anomaly detection that employ machine learning approaches often have a higher false-positive rate than prior methods relying on handcrafted signatures. As a result, ML anomaly-based systems encounter challenges in processing data and detecting real-time intrusions. To overcome these limitations, the learning process in these systems requires high-dimensional training data, resulting in more complexity and processing time than alternative methodologies [8].

In light of these existing challenges, this paper proposes an intrusion detection model that uses a GA-based feature selection method and a hybrid PSOGWO-BP algorithm. In this model, the GA-based feature selection method is first applied to the NSL-KDD dataset to select a subset of the most optimal set of features with high correlation. Then, the proposed HPSOGWO-BPNN algorithm is used to implement the model that identifies attacks with the highest detection performance. More specifically, by leveraging the strengths of PSO and GWO in hybridization, the model optimizes the initial weights and thresholds of a back-propagation Neural Network (BPNN) using a hybrid Particle Swarm Optimization (PSO) and Grey Wolf Optimization (GWO) algorithm. The significant contributions of this study are summarized as follows:

- We used a hybrid method that combines the CFSSubsetEval algorithm and a genetic algorithm to obtain a small and efficient subset of features.
- A novel hybridization approach for identifying malicious attacks is proposed based on the PSOGWO optimization algorithm and a back-propagation neural network.
- Using the proposed model, it is possible to optimize the initial weights and threshold and pass them to the back-propagation neural network for the training and prediction process.
- To better evaluate the PSOGWO-BP model’s performance, we measured the metrics of each class next to the overall metrics and compared them with other classification and optimization algorithms.

The rest of this article is organized as follows. Section 2 provides a literature review and prior results of various IDS methods obtained in recent years. Section 3 describes the datasets used in the experiment, then characterizes the hybrid feature selection technique and the method used for the proposed IDS model. Section 4 represents the experimental setup and an analysis of the results achieved by the proposed model. Finally, Section 5 contains our summaries and conclusions.

## 2. Related Works

Over the past years, researchers have used machine learning techniques to investigate intrusion detection systems and offer solutions to address the problems and limitations of conventional IDS methods [9]. Prior works have proposed various methods for identifying the data sample type for classifying cases into normal and anomaly classes. Consequently, this section examines the literature on the most prominent IDS techniques.

Dhaliwal et al. [10] proposed a hybrid network intrusion detection system using the XGBoost algorithm. They employed the NSL-KDD dataset to benchmark their proposed approach and compared their results with other algorithms. The test outcomes showed that XGBoost produces more beneficial results than Random Forest, Support Vector Machine (SVM), and Naive Bayes (NB) in terms of classification accuracy. These results show that fixing the values of the parameters of the XGboost model results in the model with the best performance. Using a similar approach, Jiang et al. [11] proposed a network intrusion detection system with a combination of PSO and the XGBoost algorithm called PSO-XGBoost. In the proposed system, they applied PSO to find the best structure of XGBoost, then the final Xgboost model was evaluated using the NSL-KDD dataset. The test outcomes demonstrate that the PSO-Xgboost model produces more useful results than regular XGboost, Bagging, and Random Forests in terms of overall classification accuracy.

Amiri et al. [12] developed an IDS approach using a feature selection technique and support vector machine (SVM) classifier. Their method uses a hybrid feature selection method based on a combination of forwarding feature selection and linear correlation coefficient methods. The authors conducted their experiments using the KDD Cup 99 dataset, comparing various feature selection methods in order to identify the impact of each set of features. The results achieved by the proposed method show that the feature selection technique is efficient in identifying various types of attacks, especially the R2L and probe attack classes. Hsu et al. [13] developed an IDS system using deep learning schemes. They combined a convolutional neural network (CNN) and Long-Short Term Memory (LSTM) layers in an approach called CNN-LSTM. The proposed system was evaluated on the NSL-KDD dataset. The results showed that the detection accuracy obtained by this method was good, and their proposed method was able to accurately classify the attack samples.

Benmessahel et al. [14] built an IDS system with a combination of the Multiverse Optimizer (MVO) and ANN algorithm. Their system’s main idea is to train a feed-forward neural network with the help of the MVO algorithm to identify new attacks. The results obtained by their system show good improvement in classification accuracy. In a similar experiment, the same group of authors introduced a new IDS by applying locust swarm optimization (LSO) for the training of a feed-forward neural network. In a follow-up study, Benmessahel et al. [15] combined their feed-forward neural network (FNN) with LSO to improve the system’s detection performance and help it to avoid common problems such as overfitting, underfitting, and becoming trapped in local optima. Then, they benchmarked the effectiveness of their approach based on the NSL-KDD and UNSW-NB15 datasets. The experiment outcomes demonstrated that the suggested model is more helpful in training ANN than other optimization algorithms, such as GA and PSO and is able to increase the detection rate of the IDS system.

Li et al. [16] presented an IDS algorithm that first selects a subset of features on the dataset using the gradual feature removal method; it selected only 19 of 41 features of the KDD dataset. Next, they developed a classifier using the clustering technique, ant colony algorithm, and SVM to classify dataset samples into normal and anomaly classes. Their experimental results showed that the proposed approach classifies over 98% of samples accurately. Wang et al. [17] proposed an intrusion detection framework using the SVM algorithm. The NSL-KDD dataset was used for validation of the method. Their empirical outcomes indicated that the suggested method produces good performance in terms of detection accuracy, and is better than other methods. However, they did clarify certain information, such as the number of training and testing samples and the statistics of the dataset they used. Moreover, SVM is not an ideal option when large datasets are involved, as analyzing massive network traffic and huge datasets can decrease its performance and increase computation costs. Lee et al. [18] introduced a new feature selection method to improve the performance of IDS methods. Their approach aims to construct a subset of best features by applying a sequential forward floating search (SFFS) algorithm on the NSLKDD dataset, then using the Random Forest classifier to classify attacks based on reduced features. The method outcomes demonstrate that feature reduction can increase the overall performance of different models, as the proposed SFFS-RF approach had a positive influence on system resource management and improved the system detection rate. Aljawarneh et al. [19] proposed a two-level hybrid intrusion detection system for achieving a high detection rate. First, their method selects the important features that positively affect accuracy using a voting algorithm with information gain. Next, it classifies attacks using selected features based on the hybrid algorithm along with the use of various classifiers such as Random Tree, REPTree, AdaBoostM1, J48, Decision Stump, and Naive Bayes. Their model was evaluated by conducting experiments on binary and multiclass classification using the NSL-KDD dataset. The experimental outcomes show that the proposed approach achieves a good detection rate while maintaining a low false-positive rate.

Consequently, according to the literature, it is evident that good classifiers used alongside dimension reduction algorithms present better performance and obtain good results in terms of classification accuracy by improving the detection rate, and additionally have shorter processing times. In this paper, therefore, we propose an IDS model based on a BPNN optimized by the PSOGWO algorithm. Then, we apply a hybrid GA-based feature selection method to eliminate irrelevant features in order to improve the overall detection rate of the model in binary and multi-class classification problems.

## 3. Methodology

This study aims to design and implement an IDS model that can effectively detect cyberattacks with high accuracy. In this section, we describe the dataset, feature selection technique, and implementation of the proposed model.

### 3.1. Data Collection

In most cases, applying a proper dataset performs an essential role in evaluating intrusion detection systems. There are various datasets, such as NSL-KDD, KDD Cup 99, ADFA, and UNSW-NB15, which can be used to evaluate IDS approaches [20]. The ADFA IDS datasets provide a representation of contemporary attack structures and tactics, and include versions for Windows and Linux [21]. UNSW-NB15 is a mix of contemporary network traffic attack behaviors that are produced both actual and synthetically, and contains nine different types of attacks [22]. The UCI machine learning repository has provided KDD Cup 99, one of the most well-known datasets for validating network security solutions. The current dataset is a revised version of the KDD CUP dataset, which excludes noisy, irrelevant, and duplicate records in order to help with performance evaluation and overcome certain significant problems with the KDD Cup 99 dataset [23].

The NSL-KDD dataset contains several training and testing datasets, including a training dataset with a 20% subset of the total training records and a full test set. In this study, we used the 20% version of the NSL-KDD training dataset, which can help reduce the cost and time of model training while increasing efficiency compared with the full version. We made three major adjustments to the NSL-KDD dataset, as follows: (i) Improving classification performance by eliminating redundant records from the KDD CUP dataset. (ii) Splitting dataset into testing and training sets with a fair number of records to provide the ability to perform experiments on the full set. (iii) Choosing a number of samples inversely proportionate to the original dataset’s record percentages on each problematic level group.

Therefore, the number of classes in the binary dataset is reduced to two main classes. The normal class is for regular records and the anomaly class is for attack records, which contain all four types of attacks. In the multi-class scenario, the number of classes is five, including all varieties of attacks such as DoS, Probe, U2R, and R2L. Table 1 presents comprehensive information about the number of instances and futures of the NSL-KDD dataset. In general, the NSL-KDD dataset is a well-known dataset employed to evaluate the various intrusion detection methods. Hence, it is used here to validate the proposed model.

### 3.2. Feature Selection

In large datasets such as NSL-KDD and KDD Cup 99, all features are not essential or necessary to develop an effective intrusion detection method. Moreover, in most situations, irrelevant and redundant features negatively impact the detection accuracy of the classification algorithm, and these irrelevant features increase the complexity and processing time of the IDS [24]. Nevertheless, feature selection is an essential part of pre-processing, which involves eliminating irrelevant and redundant features from the initial dataset and selecting the most efficient set of features in order to positively impact classification accuracy and reduce processing time [25]. Because of such benefits as quicker processing time and greater simplicity, feature selection is an essential task in developing robust intrusion detection systems. To avoid high dimensionality, a hybrid method can be used to select the efficient subset of features of the dataset; in this manner, we used a combination of the GA and CfsSubsetEval methods on the NSL-KDD dataset. Feature selection has two significant advantages. First, it makes the processing time shorter by removing redundant and irrelevant features. Second, it produces a better classification accuracy rate in comparison to that provided by a classifier without feature selection. To perform this task, the NSL-KDD dataset was evaluated using the WEKA tool in order to obtain the smallest appropriate set of features that provide the type of instances to be predicted more accurately. Investigation of 41 features of the NSL-KDD dataset determined that twenty-six features are better correlated for identifying the type of instances, and have a positive impact on the task of anomaly detection. The number of features selected from the dataset is presented in Table 2.

### 3.3. Pre-Processing Phase

To improve performance, testing, and training using the proposed model, all nominal and non-numerical values in the dataset were converted to numerical values during the pre-processing phase. All pre-processing operations performed on the dataset are described below.

1. All of the nominal and non-numerical features were replaced with unique numerical values in the testing and training sets. Certain features were replaced with unique numerical values in this step, namely, protocol type, flag, and service, all of which were identified as non-numerical features.
2. Non-numerical classes at the end of the dataset were replaced with a unique numerical value. In binary problems, the number 1 represents normal data, and the number 2 is used to represent anomalous data. In the multi-class scenario, classes were replaced in sequence by numbers from 1 to 5.
3. The features of the NSL-KDD dataset have various ranges of values, which it makes them incomparable; hence, in order to overcome this problem, the min-max normalization method was used to normalize the value of the features, with the values of all features set in the [0–1] range.

### 3.4. Particle Swarm Optimization (PSO) Algorithm

Particle swarm optimization (PSO) is an evolutionary computation swarm algorithm developed by Kennedy and Eberhart [26]. This method is a meta-heuristic algorithm that builds a simpler model inspired by the behavior and activity of birds. In PSO, each particle searches for a solution that is known as the local best, and the global best solution is then found based on the experiences of all practices. Each practice contains two features, namely, position and velocity; the position is the direction of movement, and the velocity represents a step in this movement. The velocity and position features are constantly updated during the searching process, and the iteration exits when the algorithm reaches the termination condition. The PSO algorithm implements the following five steps for optimal searching.

Step 1: Initialize the population and velocity of each particle.

Step 2: Every individual particle searches for an optimal solution with the velocity initialized in the first step. The speed of particles is based on the local and global best; in each loop, every particle obtains a solution corresponding to the local best. The optimal solution in all particles corresponds to the global best. The velocity is updated in the current step as expressed below:

(1) $$\nu_{j}^{i+1}=\omega \nu_{j}^{\left(i\right)}+\left(c_{1}\times r_{1}\left(localbest_{j}-x_{j}^{i}\right)\right)+\left(c_{2}\times r_{2}\left(globalbest_{j}-x_{j}^{i}\right)\right),\nu_{min}\leq \nu_{j}^{i}\leq \nu_{max}$$

where v(i) expresses the velocity of the v(i) particle in the ith iteration, x(i) represents the position of each particle, the coefficient of inertial weight is indicated by $w,r_{1}\;\mathrm{and}\;r_{2}$ denote numbers in the interval, and I indicates the number of repetitions.

Step 3: After the velocity of particles is measured and updated, each particle searches in the search space with a new velocity, and the fitness is computed and updated based on a fitness function.

Step 4: the local and best solution is updated for a better position based on the fitness value; the local best update is described as follows:

(2) $$X_{j}^{i+1}=X_{j}^{\left(i\right)}+\nu_{j}^{\left(i+1\right)};j=1,2,...,n$$

Step 5: The achievement and termination conditions are checked, and if the termination condition is obtained, then the searching process ends; otherwise, the process returns to Step 2.

### 3.5. Grey Wolf Optimization (GWO) Algorithm

The Grey Wolf Optimizer (GWO) is a nature-inspired optimization algorithm developed by Mirjalili et al. [27] in 2014. This algorithm mimics the social leadership hierarchy and hunting system of grey wolves to find an optimal solution. Grey wolves usually live and hunt in a group of five to twelve wolves. They are social animals, and have a strict social hierarchy. There are four types of wolves in their pack, called alpha (α), beta (β), delta (δ), and omega (ϖ). Alpha has the first position in the pack, and makes decisions such as hunting, walking time, and finding a place to sleep. The next position is beta. The beta wolves have different responsibilities, and usually help the alpha in decisions or engage in other activities; they are alpha candidates. The third type is delta. Delta wolves follow the rules and decisions of the first two types, and they are mainly responsible for protection, reconnaissance, guarding, and other duties. Finally, the last type is omega; they preserve the hierarchical arrangement’s integrity and obey the first three types of wolves. The mathematical structure of the GWO algorithm, hierarchy, and behaviors is described as follows:

(1) In the GWO algorithm, the three best solutions found are alpha (α), beta (β), and delta (δ). These three types order the omega type to explore for the best answer in the search space:

(3) $$\begin{array}{c} d=|c.x_{p}\left(t\right)-x\left(t\right)|\\ x\left(t+1\right)=x_{p}\left(t\right)-a.d. \end{array}$$

(2) The wolves should encircle the prey for hunting. The mathematical equations used to represent the encircling form of each wolf are shown below:

(4) $$\begin{array}{c} a=2l.r_{1}\\ c=2.r2. \end{array}$$

(3) The values of the *a* and *c* vectors are presented as follows.

Hunting: The alpha runs the whole hunting process. It is considered that alpha, beta, and delta wolves have better information regarding the possible prey position. Mathematically, this is formulated in the following equations:

(5) $$\begin{array}{c} \vec{d_{\alpha }}=\left|\vec{c_{1}}.\vec{x_{\alpha }}-\vec{x}\right|,\\ \vec{d_{\beta }}=\left|\vec{c_{2}}.\vec{x_{\beta }}-\vec{x}\right|,\\ \vec{d_{\delta }}=\left|\vec{c_{3}}.\vec{x_{\delta }}-\vec{x}\right|\\ \vec{x_{1}}=|\vec{x_{\alpha }}-\vec{a_{1}}.\left(\vec{d_{\alpha }}\right),\\ \vec{x_{2}}=|\vec{x_{\beta }}-\vec{a_{2}}.\left(\vec{d_{\beta }}\right),\\ \vec{x_{3}}=|\vec{x_{\delta }}-\vec{a_{3}}.\left(\vec{d_{\delta }}\right),\\ \frac{\vec{x_{1}}+\vec{x_{2}}+\vec{x_{3}}}{3}\\ \vec{a_{\left(.\right)}}=\vec{2l}.\vec{r1}-\vec{l},\\ \vec{c_{\left(.\right)}}=2.\vec{r2}. \end{array}$$

(4) Searching for prey and attacking: here, A has a random value in the range [−a, a]. The value of A decreases from 2 to 0 during the search. When A| < 1, this indicates that the wolves are forced to attack and diverge from the prey, while if A| > 1, the wolves pack should diverge and continue to search for more optimal prey. The process of searching for suitable prey is based on the locations of the α, β, and δ wolves.

### 3.6. The Hybrid PSOGWO Algorithm

The combination of the PSO and GWO algorithms was developed in [28]. The main idea of PSOGWO is to enhance the algorithm’s ability to use PSO with the capability of explore GWO in order to use the strengths of both optimization algorithms. In the process of this algorithm, the three agents’ positions are first updated; then, instead of applying standard mathematical equations, the exploitation and exploration of grey wolves is managed by an inertial constant. Mathematically, this is expressed as follows:

(6) $$\begin{array}{c} \vec{D_{\alpha }}=\left|\vec{c_{1}}.\vec{x_{\alpha }}-w*\vec{x}\right|,\\ \vec{D_{\beta }}=\left|\vec{c_{2}}.\vec{x_{\beta }}-w*\vec{x}\right|,\\ \vec{D_{\delta }}=\left|\vec{c_{3}}.\vec{x_{\delta }}-w*\vec{x}\right| \end{array}$$

For the combination of the PSO and GWO algorithms, the positions and velocity values are updated as described below:

(7) $$\nu_{j}^{k+1}=\omega *\left(\nu_{i}^{\left(k\right)}+c_{1}r_{1}\left(x_{1}-x_{i}^{k}\right)+c_{2}r_{2}\left(x_{2}-x_{i}^{k}\right)+c_{3}r_{3}\left(x_{3}-x_{i}^{k}\right)\right)$$

(8) $$X_{i}^{k+1}=X_{i}^{k}+\nu_{i}^{\left(k+1\right)}$$

### 3.7. PSOGWO-BPNN Model

**Figure 1:**
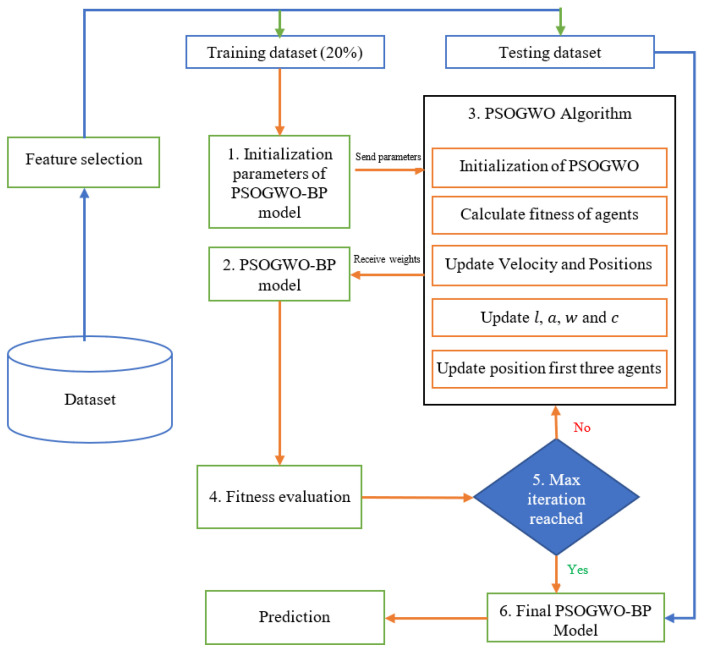
Scheme of the development of the PSOGWO-BPNN model.

This section describes the proposed PSOGWO-BP model. In this model, the PSOGWO algorithm is used to find the optimal weights and thresholds in the neural network structure. This allows the obtained weights to be initialized in the neural network in order to overcome local minimum errors; the back-propagation method is then used to train the neural network with the weights determined by the PSOGWO algorithm in order to improve further BP neural network convergence and achieve the optimal solution. The main structure of the proposed model is presented in Figure 1.

As shown in Figure 1, the proposed model contains a set of processes, which are listed as follows:

Step 1: First, it determines the topology and structure of the neural network, initializes the BP neural network, and determines the initial value and threshold, including the number of neurons and hidden layers.

Step 2: The PSOGWO algorithm first needs to initialize algorithm values, such as population size, upper and lower bound, particle positions, and velocity; then, it computes the corresponding fitness function.

Step 3: In each iteration, the PSOGWO algorithm assigns new updated weights to the artificial neural network (ANN), which evaluates and measures their fitness based on the training dataset and then returns the results to the optimization algorithm.

Step 4: The algorithm then updates certain parameters, such as the position and velocity of particles and the fitness function, then check whether it has reached the maximum number of iterations.

Step 5: Finally, the optimal weights and thresholds obtained by the PSOGWO algorithm are assigned to the BP neural network for use in the training process based on the BP algorithm, which determines whether the training results reach the termination condition. When the termination condition is reached network training stops, the trained neural network is applied to the test dataset, and the results are shown.

## 4. Experiments and Analysis of Results

This section includes the experimental setup and the investigation of the experimental outcomes of the proposed model. Section 4.1 explains the preparation and method of the experiment, Section 4.2 describes the metrics used to evaluate the proposed model’s performance, and Section 4.3 reports an analysis of the results achieved by the proposed model in order to determine its performance in detecting different types of attacks. Furthermore, the achieved results are compared with the results of other techniques.

### 4.1. Experiment Setup

To extensively study the performance of the proposed model, we conducted two sets of experiments. First, we studied the difference between the results of a number of classification algorithms and the proposed model. In the second set of experiments, we ran a comparative study between the PSOGWO algorithm and other meta-heuristic algorithms used to optimize BP neural network weights and thresholds, as well as the standard BP neural network. The algorithms used in our comparative study are PSO [26], GWO [27], SCA [29], and WOA [30]. In the implementation of these algorithms, several parameters were common and fixed to similar values. The size of the population was set to 20, the maximum number of iterations to 200, and the upper and lower bound values to (1.5,−1.5). Furthermore, the initial values of important parameters of each algorithm used in this study are provided in Table 3. All algorithms results are reported based on 20 independent runs in order to produce more reliable results. All the methods and algorithms were implemented on a computer with 16GB RAM using MATLAB R2020a software.

### 4.2. Evaluation Metrics

In the experiments used to evaluate the performance of the proposed model, we use several well-known metrics, namely, the true positive rate (TPR), false positive rate (FPR), precision, recall, F1-measure, and classification accuracy (ACC). The value of the TPR is measured by Equation (9):

(9) $$TPR=\frac{TP}{\left(P\right)}=\frac{TP}{\left(TP+FN\right)}$$

In Equation (9), *P* represents the number of anomalies and the TP denotes all attacks in the dataset which are correctly classified as anomalies. Here, *P* is equal to the value of TP and the number of records incorrectly identified as anomalies (FN). The FPR estimates the percentage of records which are incorrectly identified as anomalies. The value of FPR is measured by Equation (10):

(10) $$FPR=\frac{FP}{\left(L\right)}=\frac{FP}{\left(FP+TN\right)}$$

In Equation (10), *L* represents the total number of normal records and FP the total number of normal items in the dataset inaccurately classified as anomalies. Moreover, *L* is equal to the FP value and the number of records correctly identified as the normal class, that is, (TN). The classification accuracy (ACC) estimates the closeness between forecasted records’ evaluations and the total number of normal and anomalous records. The value of ACC is measured by Equation (11):

(11) $$ACC=\frac{\left(TP+TN\right)}{\left(P+L\right)}$$

False Negative Rate (FN) represents the percentage of anomalous records incorrectly classified as normal by the proposed model; here, *S* denotes the total number of anomalous records, while *T* represents the number of anomalies incorrectly classified as normal. The value of FN is measured using Equation (12):

(12) $$FN=\frac{T}{S}$$

Precision represents the percentage of records classified correctly as anomalies by the proposed model. It presents the model’s efficiency in detecting the correct class of records in the dataset. The value of precision is measured using Equation (13):

(13) $$Precision=\frac{TP}{\left(TP+FP\right)}$$

Recall describes the percentage of anomalous records correctly classified as anomalies, and presents the completeness. It is calculated using Equation (14):

(14) $$Recall=\frac{TP}{\left(TP+FN\right)}$$

*F-measure* is expressed as a trade-off between the precision and recall values, and indicates a harmonic mean between these two metrics. It is computed using Equation (15):

(15) $$F-measure=\frac{\left(2\times Precision\times Recall\right)}{\left(Precision+Recall\right)}$$

### 4.3. Analysis of Results

To evaluate the proposed model’s performance, in the first set of experiments we compared the proposed model’s results with different benchmark algorithms, including J48, Naive Bayes, and Random Forest, in binary and multi-class classification.

**Figure 2:**
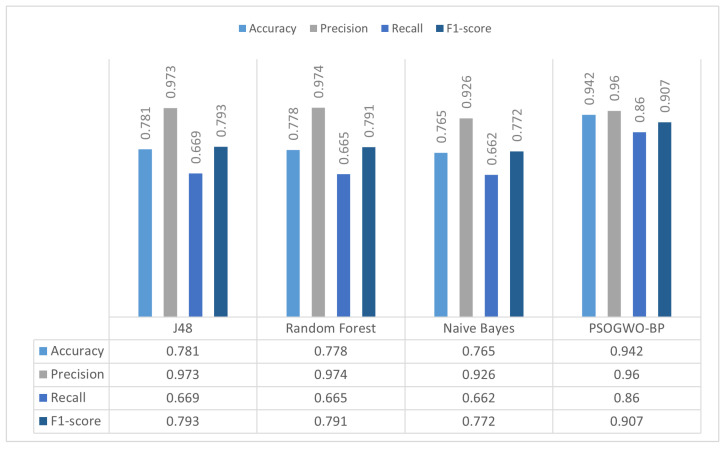
Comparison of the proposed model with other classifiers on the binary classification task.

Figure 2 presents the proposed system’s results on the comparison experiment, showing that the model obtained the highest accuracy rate of 0.942. It can be seen that the proposed model outperforms the other algorithms in terms of classification accuracy on the binary classification task. After the presented model, the J48 and Random Forest algorithms achieved the highest results; however, the gap between them and the suggested model is significant. A further multi-class classification experiment was performed for better comparison, with the results presented in Table 4.

As shown in Table 4, the proposed model is able to successfully distinguish normal items from abnormal ones, with a large gap to the other algorithms considered. Furthermore, it obtains the highest classification results in most classes, excluding only the R2L class. The other considered algorithms performed well, and the difference with the proposed model is insignificant in several classes. In the R2L class, the Random Forest algorithm produced the best result, with a difference of approximately 5% compared to the PSOGWO-BP model. In contrast, in the Probe class, the proposed model obtained the highest accuracy with 96.88%; after that, J48 obtained the highest results at 94.1%. Naive Bayes and Random Forest obtained similar results, classifying 89% of attacks in the Probe class. In the DOS class, the proposed algorithm classified 93.03% of items successfully, while J48 and Random Forest obtained 91.65% and 91.49%, respectively. The Naive Bayes classifier produced the weakest results among algorithms in this class.

**Figure 3:**
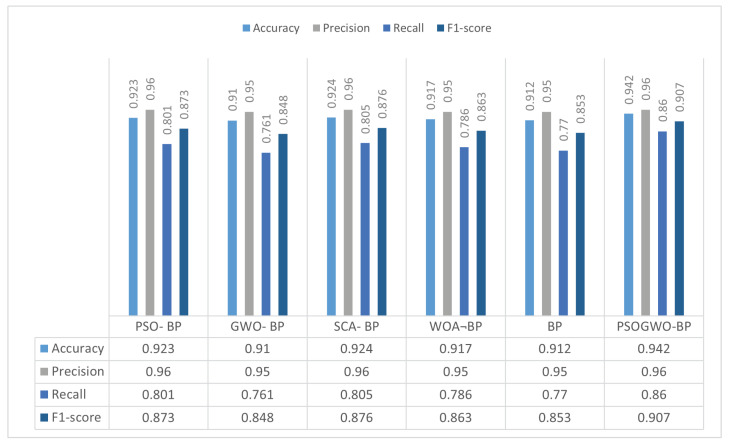
Comparison of the proposed model with other optimization algorithms in binary classification.

In the second part of the experiment, we compared the proposed model’s results with the regular Back-Propagation neural network and other metaheuristics algorithms in terms of optimizing the BP neural network’s initial weights and thresholds for binary and multi-class classification. Figure 3 presents the results achieved by the proposed model and the other metaheuristics algorithms in binary classification.

As the results show, the proposed method is more beneficial than the other techniques, achieving a higher classification accuracy and F1-score than the other algorithms in binary classification. It is able to successfully classify over 94% of items. These results indicate that the selected features perform satisfactorily with the proposed model. Next, we carried out a comparative study of multi-class classification between the achieved results of the PSOGWO algorithm and the considered metaheuristics algorithms in order to optimize the BP neural network, with results reported in Table 5.

The data in Table 5 show a better improvement in the performance of the BP neural network when using the PSOGWO algorithm compared with other metaheuristics algorithms. The PSOGWO-BP model produces more accurate results, with the lowest error rate in all classes, especially in the Normal, DoS, and U2R classes, although it has the highest error rate in the other two classes. The results obtained by all the algorithms show that while the recall value for the U2R class is not good, the precision value is high. This indicates that the actual samples predicted by the classifier all belong to the U2R class, indicating that the poor recall results are due to the small sample size. More specifically, many of the samples in the U2R class are predicted wrongly, as in other classes. In this case, the F1-score can represent a better balance between precision and recall, as indicated in Table 5. For the Probe class, the accuracy of the SCA-BP algorithm is better than the proposed model, although the difference between them is only about 0.30%, which is not a massive difference. On the other hand, PSO-BP has the highest accuracy on the R2L class, where its scores approximately 2% higher than PSOGWO-BP. Compared with the regular BP neural network, the proposed model achieves the highest accuracy in all classes except the Normal class, in which the BP algorithm produces better results, achieving 0.70% higher accuracy than PSOGWO-BP.

## 5. Conclusions and Future Work

In this research, we have aimed to develop a hybrid anomaly-based intrusion detection model with high classification accuracy. In our model, a hybrid feature selection method is suggested for selecting the most correlated features based on the GA and CfsSubsetEval algorithms in order to reduce complexity and processing time while increasing model performance. We optimized the BP neural network’s initial weights and threshold using a hybrid combination of the PSO and GWO algorithms, then applied the results to the selected features. Finally, we evaluated the proposed model’s performance against other conventional algorithms in binary and multi-class classification tasks on the NSL-KDD dataset. The test outcomes show that the PSOGWO-BP model can help to identify different types of cyberattacks, and produces better results than the other considered algorithms. Furthermore, this paper’s findings show that there is potential for the use of optimization algorithms in reducing and overcoming the disadvantages of BP neural networks, such as slow convergence rates and local minimum errors. The current model can be used to solve other binary and multiclass classification problems as well.

Moreover, there are several promising future research directions worth exploring. A limitation of the NSL-KDD dataset is the presence of minority attack cases such as Remote-to-Local (R2L) and User-to-Root (U2R), which are known to impact classifier performance and lead to an overall increase in misclassification rate and false positives. In future work, we suggest that the class imbalance problem can be addressed using traditional methods (e.g., oversampling and random undersampling) and hybrid deep learning techniques. Furthermore, the cases can be augmented using synthetic data generated from heterogeneous testbeds that mimic real-world network environments. Because the IDS model was validated in isolation, future research on heterogeneous testbeds might concentrate on deploying the model against particular phases of multi-stage network attacks. For real-time deployment, key performance indicators such as CPU utilization, latency, detection rate, and inference rate can be explored to evaluate the performance and scalability of the model. In this context, it would be worthwhile to investigate the performance of various other models and deep learning classifiers that have proven be suitable for nonlinear and high-dimensional datasets.

## Figures and Tables

**Table 1:** Description of the dataset.

	Dataset Records	
	Train	Test
Normal	13,423	9711
Dos	9206	7458
Probe	2365	2421
U2R	9	200
R2L	191	2754
Total	25,194	22,544

**Table 2:** List of selected features.

	Feature Name		Feature Name
1	duration	14	count
2	protocol_type	15	serror_rate
3	flag	16	srv_serror_rate
4	dst_bytes	17	srv_rerror_rate
5	wrong_fragment	18	same_srv_rate
6	urgent	19	diff_srv_rate
7	num_failed_logins	20	dst_host_count
8	num_compromised	21	dst_host_srv_count
9	root_shell	22	dst_host_same_srv_rate
10	num_root	23	dst_host_srv_diff_host_rate
11	num_shells	24	dst_host_serror_rate
12	is_host_login	25	dst_host_srv_serror_rate
13	is_guest_login	26	dst_host_rerror_rate

**Table 3:** Parameter settings of optimization algorithms.

	Parameters	Value
PSO	inertia w	1
	Acceleration constants	(1.5, 2.0)
GWO	a	Linearly decreased from 2 to 0
SCA	a	1
	*r*_1_	Linearly decreased from a to 0
WOA	a	Linearly decreased from 2 to 0
	*a*_2_	Linearly decreased from −1 to −2

**Table 4:** Comparison of the performance of the proposed model and different classification algorithms in detecting of different types of attacks.

Algorithm	Class	Accuracy	Precision	Recall	F1
J48	Normal	78.82	0.79	0.95	0.86
	Dos	91.65	0.79	0.95	0.86
	Probe	94.99	0.66	0.84	0.74
	U2R	99.11	0.01	0.50	0.02
	R2L	89.51	0.14	0.99	0.25
Random Forest	Normal	77.08	0.97	0.66	0.79
	Dos	91.49	0.77	0.96	0.86
	Probe	89.14	0.11	0.99	0.20
	U2R	99.11	0.005	0.33	0.009
	R2L	95.63	0.69	0.88	0.77
Naive Bayes	Normal	81.72	0.83	0.76	0.80
	Dos	87.13	0.68	0.91	0.78
	Probe	89.39	0.77	0.50	0.61
	U2R	89.46	0.32	0.02	0.05
	R2L	88.65	0.10	0.77	0.18
PSOGWO-BP	Normal	83.82	0.74	0.96	0.84
	Dos	93.03	0.91	0.88	0.89
	Probe	96.88	0.89	0.81	0.85
	U2R	99.13	0.64	0.005	0.08
	R2L	89.23	0.95	0.13	0.22

**Table 5:** Comparison of the performance of the proposed model and different metaheuristics algorithms in optimizing the BP neural network.

Algorithm	Class	Accuracy	Precision	Recall	F1
PSO-BP	Normal	79.41	0.68	0.97	0.80
	Dos	90.53	0.95	0.76	0.84
	Probe	95.15	0.84	0.68	0.75
	U2R	99.11	0.33	0.005	0.009
	R2L	91.07	0.93	0.29	0.44
GWO-BP	Normal	78.02	0.67	0.95	0.79
	Dos	90.68	0.93	0.78	0.85
	Probe	96.55	0.88	0.79	0.83
	U2R	99.11	0.0	0.0	0.0
	R2L	89.56	0.94	0.15	0.27
SCA-BP	Normal	80.72	0.70	0.97	0.81
	Dos	90.62	0.93	0.77	0.85
	Probe	97.19	0.89	0.84	0.87
	U2R	99.11	0.0	0.0	0.0
	R2L	90.24	0.96	0.21	0.34
WOA-BP	Normal	79.52	0.69	0.97	0.80
	Dos	90.34	0.92	0.77	0.84
	Probe	96.33	0.90	0.74	0.81
	U2R	99.11	0.0	0.0	0.0
	R2L	90.22	0.97	0.21	0.34
BP	Normal	81.25	0.71	0.97	0.82
	Dos	92.72	0.94	0.83	0.88
	Probe	95.97	0.86	0.74	0.80
	U2R	99.11	0.0	0.0	0.0
	R2L	89.93	0.97	0.18	0.31
PSOGWO-BP	Normal	83.82	0.74	0.96	0.84
	Dos	93.03	0.91	0.88	0.89
	Probe	96.88	0.89	0.81	0.85
	U2R	99.13	0.64	0.005	0.08
	R2L	89.23	0.95	0.13	0.22

## Data Availability

Not applicable.
